# Supplementary material for: Mitogenomic Phylogeny of Tonnoidea Suter, 1913 (1825) (Gastropoda: Caenogastropoda)
Source: Animals (Basel). 2023 Oct 27;13(21):3342. doi: 10.3390/ani13213342 (PMC10649890; doi:10.3390/ani13213342)
Supplement: Supplementary file 1 [file animals-13-03342-s001.zip › animals-2623138-supplementary.pdf]

Table S1. Best fit partitions and substitution models.

	Set Partition (Conducted by PartitionFinder 2)	Best Model for BI (Selected by PartitionFinder 2)
Best Partition CDS genes (BIC=63,885)	*atp6-8*	MTMAM+I+G
	cox1-2-3	MTART+I+G
	cob	MTART+I+G
	nad1-2-3-4-4L-5-6	MTMAM+I+G+F
Best Partition to rRNA genes (BIC= 27648.25)	*rrnS-L*	GTR+I+G

Table S2. Basic composition of mitochondrial genome of *Monoplex pilearis*.

Gene	Strand	Location	Size（bp）	Start codon	Stop codon	Intergenic nucleotides
Cox3	H	1-780	780	ATG	TAA	13
tRNA-Lys	H	794-868	75			2
tRNA-Ala	H	871-940	70			33
tRNA-Arg	H	974-1042	69			0
tRNA-Asn	H	1043-1109	67			13
tRNA-Ile	H	1123-1194	72			2
Nad3	H	1197-1550	354	ATG	TAA	-1
tRNA-Ser	H	1550-1619	70			-1
Nad2	H	1619-2677	1059	ATG	TAA	154
Cox1	H	2832-4367	1536	ATG	TAA	15
Cox2	H	4383-5069	687	ATG	TAA	-3
tRNA-Asp	H	5067-5136	70			0
Atp8	H	5137-5295	159	ATG	TAA	2
Atp6	H	5298-5993	696	ATG	TAA	34
tRNA-Met	L	6028-6097	70			6
tRNA-Tyr	L	6104-6174	71			-1
tRNA-Cys	L	6174-6239	66			-2
tRNA-Trp	L	6238-6307	70			-3
tRNA-Gln	L	6305-6374	70			1
tRNA-Gly	L	6376-6444	69			1
tRNA-Glu	L	6446-6515	70			0
12s	H	6516-7494	979			0
tRNA-Val	H	7495-7564	70			0
16s	H	7565-8932	1368			0
tRNA-Leu	H	8933-9002	70			0
tRNA-Leu	H	9003-9073	71			-1
Nad1	H	9073-10003	941	ATG	TA	10
tRNA-Pro	H	10014-10080	67			2
Nad6	H	10083-10583	501	ATG	TAG	5
Cytb	H	10589-11728	1140	ATG	TAA	10
tRNA-Ser	H	11739-11805	67			1
tRNA-Thr	L	11807-11872	66			9
Nad4L	H	11882-12178	297	ATG	TAG	28
Nad4	H	12207-13545	1339	ATG	TAA	6
tRNA-His	H	13552-13617	66			0
Nad5	H	13618-15339	1722	ATG	TAA	4
tRNA-Phe	H	15344-15412	69			150

“H” indicates the heavy strand while “L” indicates the light strand.

Table S3. Basic composition of mitochondrial genome of *Tutufa bubo*.

Gene	Strand	Location	Size（bp）	Start codon	Stop codon	Intergenic nucleotides
Cox3	H	1-780	780	ATG	TAG	11
tRNA-Lys	H	792-864	73			40
tRNA-Ala	H	905-973	69			8
tRNA-Arg	H	982-1050	69			0
tRNA-Asn	H	1051-1116	66			21
tRNA-Ile	H	1138-1209	72			1
Nad3	H	1211-1564	354	ATG	TAA	0
tRNA-Ser	H	1565-1634	70			-1
Nad2	H	1634-2692	1059	ATG	TAA	4
Cox1	H	2697-4232	1536	ATG	TAG	14
Cox2	H	4247-4933	687	ATG	TAA	-3
tRNA-Asp	H	4931-5000	70			0
Atp8	H	5001-5159	159	ATG	TAA	2
Atp6	H	5162-5857	696	ATG	TAG	40
tRNA-Met	L	5898-5964	67			11
tRNA-Tyr	L	5976-6041	66			8
tRNA-Cys	L	6050-6113	64			-2
tRNA-Trp	L	6112-6180	69			-2
tRNA-Gln	L	6179-6245	67			-1
tRNA-Gly	L	6245-6314	70			-2
tRNA-Glu	L	6313-6383	71			0
12s	H	6384-7355	972			0
tRNA-Val	H	7356-7425	70			0
16s	H	7426-8819	1394			0
tRNA-Leu	H	8820-8890	71			-1
tRNA-Leu	H	8890-8960	71			-1
Nad1	H	8960-9901	942	ATG	TAA	0
tRNA-Pro	H	9902-9969	68			1
Nad6	H	9971-10471	501	ATG	TAA	24
Cytb	H	10496-11635	1140	ATG	TAA	4
tRNA-Ser	H	11640-11706	67			2
tRNA-Thr	L	11709-11774	66			13
Nad4L	H	11788-12084	297	ATG	TAG	10
Nad4	H	12095-13451	1357	ATA	TAA	16
tRNA-His	H	13468-13532	65			0
Nad5	H	13533-15254	1722	ATG	TAG	-1
tRNA-Phe	H	15254-15321	68			151

“H” indicates the heavy strand while “L” indicates the light strand.

Table S4. Basic composition of mitochondrial genome of *Gyrineum natator*.

Gene	Strand	Location	Size（bp）	Start codon	Stop codon	Intergenic nucleotides
Cox3	H	1-780	780	ATG	TAA	12
tRNA-Lys	H	793-861	69			10
tRNA-Ala	H	872-940	69			9
tRNA-Arg	H	950-1020	71			2
tRNA-Asn	H	1023-1090	68			11
tRNA-Ile	H	1102-1170	69			3
Nad3	H	1174-1527	354	ATG	TAA	-2
tRNA-Ser	H	1526-1595	70			-1
Nad2	H	1595-2653	1059	ATG	TAA	4
Cox1	H	2658-4193	1536	ATG	TAA	16
Cox2	H	4210-4896	687	ATG	TAA	-2
tRNA-Asp	H	4895-4963	69			0
Atp8	H	4964-5122	159	ATG	TAA	2
Atp6	H	5125-5820	696	ATG	TAA	39
tRNA-Met	L	5860-5930	71			1
tRNA-Tyr	L	5932-5999	68			6
tRNA-Cys	L	6006-6070	65			-2
tRNA-Trp	L	6069-6136	68			-2
tRNA-Gln	L	6135-6199	65			0
tRNA-Gly	L	6200-6268	69			0
tRNA-Glu	L	6269-6339	71			0
12s	H	6340-7295	956			0
tRNA-Val	H	7296-7363	68			0
16s	H	7364-8747	1384			0
tRNA-Leu	H	8748-8816	69			0
tRNA-Leu	H	8817-8885	69			0
Nad1	H	8886-9827	942	ATG	TAA	1
tRNA-Pro	H	9829-9896	68			1
Nad6	H	9898-10392	495	ATG	TAG	23
Cytb	H	10416-11555	1140	ATG	TAA	4
tRNA-Ser	H	11560-11627	68			1
tRNA-Thr	L	11629-11696	68			9
Nad4L	H	11706-12002	297	ATG	TAG	-7
Nad4	H	11996-13369	1374	ATG	TAA	6
tRNA-His	H	13376-13441	66			0
Nad5	H	13442-15163	1722	ATG	TAG	-1
tRNA-Phe	H	15163-15231	69			354

“H” indicates the heavy strand while “L” indicates the light strand.

Table S5. Basic composition of mitochondrial genome of *Tonna sulcosa*.

Gene	Strand	Location	Size（bp）	Start codon	Stop codon	Intergenic nucleotides
Cox3	H	1-780	780	ATG	TAG	11
tRNA-Lys	H	792-864	73			13
tRNA-Ala	H	878-945	68			50
tRNA-Arg	H	996-1064	69			4
tRNA-Asn	H	1069-1136	68			27
tRNA-Ile	H	1164-1236	73			2
Nad3	H	1239-1592	354	ATG	TAG	6
tRNA-Ser	H	1599-1666	68			0
Nad2	H	1595-2653	1059	ATG	TAA	4
Cox1	H	2738-4273	1536	ATG	TAA	17
Cox2	H	4291-4977	687	ATG	TAA	-3
tRNA-Asp	H	4975-5044	70			0
Atp8	H	5045-5203	159	ATG	TAA	25
Atp6	H	5229-5924	696	ATG	TAA	37
tRNA-Met	L	5962-6031	70			6
tRNA-Tyr	L	6038-6105	68			10
tRNA-Cys	L	6116-6181	66			-2
tRNA-Trp	L	6180-6248	69			-2
tRNA-Gln	L	6246-6314	69			0
tRNA-Gly	L	6315-6381	67			-1
tRNA-Glu	L	6381-6453	73			0
12s	H	6454-7414	961			0
tRNA-Val	H	7415-7482	68			0
16s	H	7483-8879	1397			0
tRNA-Leu	H	8880-8948	69			0
tRNA-Leu	H	8949-9019	71			-1
Nad1	H	9019-9960	942	ATG	TAG	0
tRNA-Pro	H	9961-10029	69			1
Nad6	H	10031-10531	501	ATG	TAA	9
Cytb	H	10541-11680	1140	ATG	TAA	5
tRNA-Ser	H	11686-11750	65			9
tRNA-Thr	L	11760-11829	70			7
Nad4L	H	11837-12133	297	ATG	TAG	-7
Nad4	H	12127-13503	1377	GTG	TAA	12
tRNA-His	H	13516-13585	70			-1
Nad5	H	13585-15306	1722	ATG	TAG	0
tRNA-Phe	H	15307-15375	69			313

“H” indicates the heavy strand while “L” indicates the light strand.

Table S6. Basic composition of mitochondrial genome of *Lotoria lotoria.*

Gene	Strand	Location	Size（bp）	Start codon	Stop codon	Intergenic nucleotides
Cox3	H	1-780	780	ATG	TAA	15
tRNA-Lys	H	796-869	74			10
tRNA-Ala	H	880-949	70			19
tRNA-Arg	H	969-1037	69			0
tRNA-Asn	H	1038-1105	68			15
tRNA-Ile	H	1121-1190	70			4
Nad3	H	1195-1548	354	ATG	TAG	0
tRNA-Ser	H	1549-1616	68			0
Nad2	H	1617-2675	1059	ATG	TAG	6
Cox1	H	2682-4217	1536	ATG	TAA	15
Cox2	H	4233-4919	687	ATG	TAA	-3
tRNA-Asp	H	4917-4986	70			0
Atp8	H	4987-5145	159	ATG	TAA	2
Atp6	H	5148-5846	699	ATG	TAA	33
tRNA-Met	L	5880-5948	69			3
tRNA-Tyr	L	5952-6022	71			7
tRNA-Cys	L	6030-6095	66			-2
tRNA-Trp	L	6094-6163	70			-2
tRNA-Gln	L	6162-6229	68			2
tRNA-Gly	L	6232-6299	68			6
tRNA-Glu	L	6306-6376	71			0
12s	H	6377-7336	960			0
tRNA-Val	H	7337-7403	67			0
16s	H	7404-8764	1361			0
tRNA-Leu	H	8765-8834	70			0
tRNA-Leu	H	8835-8903	69			0
Nad1	H	8904-9844	941	ATG	TA	0
tRNA-Pro	H	9845-9913	69			1
Nad6	H	9915-10415	501	ATG	TAA	5
Cytb	H	10421-11560	1140	ATG	TAA	6
tRNA-Ser	H	11567-11631	65			2
tRNA-Thr	L	11634-11700	67			9
Nad4L	H	11710-12006	297	ATG	TAG	-7
Nad4	H	12000-13373	1374	ATG	TAA	5
tRNA-His	H	13379-13443	65			0
Nad5	H	13444-15165	1722	ATG	TAA	13
tRNA-Phe	H	15179-15246	68			575

“H” indicates the heavy strand while “L” indicates the light strand.

Table S7. Basic composition of mitochondrial genome of *Phalium glaucum.*

Gene	Strand	Location	Size（bp）	Start codon	Stop codon	Intergenic nucleotides
Cox3	H	1-780	780	ATG	TAA	14
tRNA-Lys	H	795-863	69			14
tRNA-Ala	H	878-945	68			6
tRNA-Arg	H	952-1020	69			14
tRNA-Asn	H	1035-1105	71			1
tRNA-Ile	H	1107-1176	70			4
Nad3	H	1181-1534	354	ATG	TAA	3
tRNA-Ser	H	1538-1607	70			-1
Nad2	H	1607-2665	1059	ATG	TAA	8
Cox1	H	2674-4209	1536	ATG	TAA	21
Cox2	H	4231-4917	687	ATG	TAA	-3
tRNA-Asp	H	4915-4984	70			-1
Atp8	H	4984-5142	159	ATG	TAA	18
Atp6	H	5161-5856	696	ATG	TAA	40
tRNA-Met	L	5897-5965	69			15
tRNA-Tyr	L	5981-6049	69			9
tRNA-Cys	L	6059-6122	64			-2
tRNA-Trp	L	6121-6188	68			1
tRNA-Gln	L	6190-6251	62			5
tRNA-Gly	L	6257-6324	68			6
tRNA-Glu	L	6331-6402	72			0
12s	H	6403-7370	968			0
tRNA-Val	H	7371-7441	71			0
16s	H	7442-8849	1408			0
tRNA-Leu	H	8850-8918	69			0
tRNA-Leu	H	8919-8987	69			0
Nad1	H	8988-9929	942	ATG	TAA	0
tRNA-Pro	H	9930-9998	69			3
Nad6	H	10002-10502	501	ATG	TAA	11
Cytb	H	10514-11653	1140	ATG	TAA	8
tRNA-Ser	H	11662-11727	66			-2
tRNA-Thr	L	11726-11793	68			7
Nad4L	H	11801-12097	297	ATG	TAG	-7
Nad4	H	12091-13464	1374	ATG	TAG	14
tRNA-His	H	13479-13543	65			0
Nad5	H	13544-15265	1722	ATG	TAA	-1
tRNA-Phe	H	15265-15332	68			611

“H” indicates the heavy strand while “L” indicates the light strand.

Table S8. Basic composition of mitochondrial genome of *Semicassis bisulcate.*

Gene	Strand	Location	Size（bp）	Start codon	Stop codon	Intergenic nucleotides
Cox3	H	1-780	780	ATG	TAA	15
tRNA-Lys	H	796-870	75			12
tRNA-Ala	H	883-951	69			3
tRNA-Arg	H	955-1023	69			2
tRNA-Asn	H	1026-1092	67			3
tRNA-Ile	H	1096-1164	69			4
Nad3	H	1169-1522	354	ATG	TAA	6
tRNA-Ser	H	1529-1598	70			-1
Nad2	H	1598-2656	1059	ATG	TAA	9
Cox1	H	2666-4201	1536	ATG	TAA	17
Cox2	H	4219-4905	687	ATG	TAA	-3
tRNA-Asp	H	4903-4972	70			-1
Atp8	H	4972-5130	159	ATG	TAA	14
Atp6	H	5145-5840	696	ATG	TAA	34
tRNA-Met	L	5875-5941	67			17
tRNA-Tyr	L	5959-6026	68			10
tRNA-Cys	L	6037-6100	64			-2
tRNA-Trp	L	6099-6176	69			3
tRNA-Gln	L	6171-6232	62			1
tRNA-Gly	L	6234-6304	71			0
tRNA-Glu	L	6305-6375	71			0
12s	H	6376-7339	964			0
tRNA-Val	H	7340-7406	67			0
16s	H	7407-8789	1383			0
tRNA-Leu	H	8790-8858	69			4
tRNA-Leu	H	8863-8931	69			0
Nad1	H	8932-9873	942	ATG	TAA	0
tRNA-Pro	H	9874-9941	68			3
Nad6	H	9945-10445	501	ATG	TAA	16
Cytb	H	10462-11601	1140	ATG	TAA	7
tRNA-Ser	H	11609-11677	69			-3
tRNA-Thr	L	11675-11741	67			9
Nad4L	H	11751-12047	297	ATG	TAG	-7
Nad4	H	12041-13414	1374	ATG	TAA	19
tRNA-His	H	13434-13499	66			0
Nad5	H	13500-15221	1722	ATG	TAA	-1
tRNA-Phe	H	15221-15289	69			544

“H” indicates the heavy strand while “L” indicates the light strand.

Table S9. Basic composition of mitochondrial genome of *Phalium flammiferum.*

Gene	Strand	Location	Size（bp）	Start codon	Stop codon	Intergenic nucleotides
Cox3	H	1-780	780	ATG	TAA	15
tRNA-Lys	H	796-865	70			18
tRNA-Ala	H	884-951	68			6
tRNA-Arg	H	958-1028	71			11
tRNA-Asn	H	1040-1110	71			1
tRNA-Ile	H	1112-1181	70			4
Nad3	H	1186-1539	354	ATG	TAA	2
tRNA-Ser	H	1542-1611	70			-1
Nad2	H	1611-2669	1059	ATG	TAG	8
Cox1	H	2678-4213	1536	ATG	TAA	19
Cox2	H	4233-4919	687	ATG	TAA	-3
tRNA-Asp	H	4917-4986	70			-1
Atp8	H	4986-5144	159	ATG	TAA	16
Atp6	H	5161-5856	696	ATG	TAA	46
tRNA-Met	L	5903-5970	68			15
tRNA-Tyr	L	5986-6053	68			11
tRNA-Cys	L	6065-6129	65			-2
tRNA-Trp	L	6128-6195	68			1
tRNA-Gln	L	6197-6258	62			2
tRNA-Gly	L	6261-6331	71			0
tRNA-Glu	L	6332-6402	71			0
12s	H	6403-7382	980			0
tRNA-Val	H	7383-7452	70			0
16s	H	7453-8862	1410			0
tRNA-Leu	H	8863-8931	69			0
tRNA-Leu	H	8932-9000	69			0
Nad1	H	9001-9942	942	ATG	TAA	0
tRNA-Pro	H	9943-10011	69			3
Nad6	H	10015-10515	501	ATG	TAA	13
Cytb	H	10529-11668	1140	ATG	TAA	8
tRNA-Ser	H	11677-11742	66			-2
tRNA-Thr	L	11741-11809	69			6
Nad4L	H	11816-12112	297	ATG	TAG	-7
Nad4	H	12106-13479	1374	ATG	TAA	11
tRNA-His	H	13491-13555	65			0
Nad5	H	13556-15277	1722	ATG	TAA	-1
tRNA-Phe	H	15277-15345	69			599

“H” indicates the heavy strand while “L” indicates the light strand.

Table S10. Basic composition of mitochondrial genome of *Tutufa rubeta.*

Gene	Strand	Location	Size（bp）	Start codon	Stop codon	Intergenic nucleotides
Cox3	H	1-780	780	ATG	TAG	11
tRNA-Lys	H	792-865	74			38
tRNA-Ala	H	904-972	69			9
tRNA-Arg	H	982-1050	69			1
tRNA-Asn	H	1052-1117	66			27
tRNA-Ile	H	1145-1214	70			2
Nad3	H	1217-1570	354	ATG	TAA	-1
tRNA-Ser	H	1570-1639	70			-1
Nad2	H	1639-2697	1059	ATG	TAA	3
Cox1	H	2701-4236	1536	ATG	TAA	15
Cox2	H	4252-4938	687	ATG	TAA	-3
tRNA-Asp	H	4936-5005	70			0
Atp8	H	5006-5164	159	ATG	TAA	2
Atp6	H	5167-5862	696	ATG	TAG	40
tRNA-Met	L	5903-5968	66			9
tRNA-Tyr	L	5978-6047	70			6
tRNA-Cys	L	6054-6119	66			-2
tRNA-Trp	L	6118-6186	69			-1
tRNA-Gln	L	6186-6250	65			1
tRNA-Gly	L	6252-6319	68			0
tRNA-Glu	L	6320-6390	71			0
12s	H	6391-7359	969			0
tRNA-Val	H	7360-7428	69			0
16s	H	7429-8824	1396			0
tRNA-Leu	H	8825-8893	69			0
tRNA-Leu	H	8894-8964	71			-1
Nad1	H	8964-9905	942	ATG	TAA	0
tRNA-Pro	H	9906-9974	69			1
Nad6	H	9976-10476	501	ATG	TAA	24
Cytb	H	10501-11640	1140	ATG	TAA	5
tRNA-Ser	H	11646-11710	65			3
tRNA-Thr	L	11714-11779	66			13
Nad4L	H	11793-12089	297	ATG	TAG	-7
Nad4	H	12083-13456	1374	ATG	TAA	15
tRNA-His	H	13472-13537	66			0
Nad5	H	13538-15259	1722	ATG	TAG	-1
tRNA-Phe	H	15259-15326	68			654

“H” indicates the heavy strand while “L” indicates the light strand.
